# *Moraxella catarrhalis* phase-variable loci show differences in expression during conditions relevant to disease

**DOI:** 10.1371/journal.pone.0234306

**Published:** 2020-06-18

**Authors:** Aimee Tan, Luke V. Blakeway, Taha, Yuedong Yang, Yaoqi Zhou, John M. Atack, Ian R. Peak, Kate L. Seib

**Affiliations:** 1 Institute for Glycomics, Griffith University, Gold Coast, Queensland, Australia; 2 School of Medical Science, Griffith University, Gold Coast, Queensland, Australia; Ross University School of Medicine, DOMINICA

## Abstract

*Moraxella catarrhalis* is a human-adapted, opportunistic bacterial pathogen of the respiratory mucosa. Although asymptomatic colonization of the nasopharynx is common, *M*. *catarrhalis* can ascend into the middle ear, where it is a prevalent causative agent of otitis media in children, or enter the lower respiratory tract, where it is associated with acute exacerbations of chronic obstructive pulmonary disease in adults. Phase variation is the high frequency, random, reversible switching of gene expression that allows bacteria to adapt to different host microenvironments and evade host defences, and is most commonly mediated by simple DNA sequence repeats. Bioinformatic analysis of five closed *M*. *catarrhalis* genomes identified 17 unique simple DNA sequence repeat tracts that were variable between strains, indicating the potential to mediate phase variable expression of the associated genes. Assays designed to assess simple sequence repeat variation under conditions mimicking host infection demonstrated that phase variation of *uspA1* (ubiquitous surface protein A1) from high to low expression occurs over 72 hours of biofilm passage, while phase variation of *uspA2* (ubiquitous surface protein A2) to high expression variants occurs during repeated exposure to human serum, as measured by mRNA levels. We also identify and confirm the variable expression of two novel phase variable genes encoding a Type III DNA methyltransferase (*modO*), and a conserved hypothetical permease (MC25239_RS00020). These data reveal the repertoire of phase variable genes mediated by simple sequence repeats in *M*. *catarrhalis* and demonstrate that modulation of expression under conditions mimicking human infection is attributed to changes in simple sequence repeat length.

## Introduction

*Moraxella catarrhalis* is a Gram negative, human-adapted, opportunistic bacterial pathogen of the respiratory tract. While commonly isolated from the nasopharynx as an asymptomatic colonizer [1], *M*. *catarrhalis* is also a prevalent aetiological agent of otitis media (OM) in children and exacerbations of chronic obstructive pulmonary disease (COPD) in the elderly [2]. OM is the most common bacterial infectious disease of childhood, and is particularly prevalent in children under five in Oceania [3], with Indigenous Australian children among the most severely affected [4]. Approximately 20% of children suffer recurring infections [5], and associated complications such as repeated tympanic membrane perforation lead to acute or chronic hearing loss [6, 7]. COPD is the fourth most common cause of death worldwide [8] and repeated exacerbations due to bacterial infections lead to progressive loss of lung function and dramatically increase the risk of mortality [9]. *M*. *catarrhalis* infection accounts for approximately 20% of cases of OM [10] and 10% of exacerbations of COPD [11]. Despite the significant burden of *M*. *catarrhalis* associated disease, no proposed vaccine candidates have progressed to clinical trial [12]. The development of a vaccine against *M*. *catarrhalis* has been hindered by the lack of a suitable animal model, identification of correlates of protection, and identification of vaccine candidates that are immunogenic, conserved, and stably expressed [12]. It is this last feature that this study primarily addresses, as a number of respiratory pathogens possess a highly mutable genome, which contributes to their virulence and complicates selection of stably expressed vaccine candidates.

Phase variation is the high frequency, random, reversible switching of gene expression that allows bacteria to adapt to different host microenvironments and evade host defences, and is often mediated by simple DNA simple sequence repeats (SSRs) [13]. Switching rates of phase variable genes are approximately one million times more frequent than the background rate of mutations (i.e., phase variation switching occurs at a rate of 10^−2^ to 10^−5^ compared with point mutation rates of ∼10^−8^ to 10^−11^ for the spontaneous acquisition of antibiotic resistance) [13]. In both cases, mutations occur independently of their utility, and the environment selects for variants that facilitate bacterial growth and/or survival. To date, six loci containing SSRs have been identified in *M*. *catarrhalis*; these include four genes encoding outer membrane proteins (*mid/hag*, *uspA1* and *uspA2*, or the mutually exclusive *uspA2H* variant), and two encoding cytoplasmic localized Type III DNA methyltransferases (*modM* and *modN*) [reviewed in 14].

UspA1 is involved in biofilm formation [15], and adherence to multiple epithelial cell types [16, 17] and extracellular matrix components [18, 19]. Reversible gain or loss of repeat units in a G_(n)_ SSR tract located upstream of the *uspA1* open reading frame (ORF) alters transcription of the gene, resulting in a graded switching of expression (high-to-low) that alters the ability of *M*. *catarrhalis* to adhere to epithelial cells *in vitro* [20]. Similarly, phase variation of *uspA2* occurs at the transcriptional level through variation in length of a 5′-AGAT_(n)_-3′ tetranucleotide SSR upstream of the *uspA2* ORF, and the length of the *uspA2* repeat tract affects resistance of *M*. *catarrhalis* to complement mediated killing [21]. In the other identified phase variable genes in *M*. *catarrhalis*, SSRs are located within the ORF close to the 5′ end and cause ‘on-off’ switching of gene expression. *mid/hag* is involved in serum resistance and binding to vibronectin [21], and agglutination of *M*. *catarrhalis* cells, red blood cells and immunoglobulin D binding [22, 23]. Phase variation of *mid/hag* is mediated by a G_(n)_ SSR, with on-off switching altering auto-aggregation and adherence [23, 24]. The *uspA2H* ORF contains a A_(n)_ SSR that alters auto-aggregation, serum resistance, and adherence phenotypes [25]. We have previously shown that 5′-CAAC_(n)_-3′ tetranucleotide SSRs are responsible for phase variation of both the *modM* and *modN* genes [26], and differential methylation of the genome caused by ModM phase variation epigenetically regulates the switching of expression of multiple genes in a phasevarion [27, 28], further complicating the identification of stably expressed genes in *M*. *catarrhalis*. Further to this, we have also identified a third potentially phase variable Type III DNA methyltransferase, *modO*, which contains a 5′-CAACG_(n)_-3′ pentanucleotide repeat tract upstream of its ORF [29]. However, analysis of *modO* expression has not been described.

Comparison with other human-restricted respiratory tract mucosal pathogens suggests that *M*. *catarrhalis* contains relatively few phase variable genes. For example, *Neisseria meningitidis* contains as many as 83 putatively phase variable genes [30–32], including genes that encode outer membrane proteins, e.g., *porA* [33] and *opc* [34], genes involved in lipooligosaccharide (LOS) synthesis [35], and the *mod* methyltransferases [36, 37]. In *Haemophilus influenzae*, at least nineteen predicted phase variable genes have been identified [38], including LOS synthesis [39–41], outer membrane proteins Hia [42] and HMW [43], as well the *modA* methyltransferase that is also found in *Neisseria* species [44–47]. Many of the studied phase variable genes are virulence factors and/or vaccine candidates [48, 49]. Whilst phase variable candidates can be used in vaccines (for example, NadA in Bexsero [50–52]), there is the possibility of vaccine evasion due to phase variation. However, it may be possible to use phase variable proteins in vaccines in combination with other stably expressed determinants, or if the phase-variable candidate(s) are required at key stages during colonisation or disease.

In order to better understand phase variable gene expression, here we report the repertoire of putatively phase variable genes in *M*. *catarrhalis* that are associated with SSRs and investigate the expression of three previously identified and three novel phase variable gene candidates. We also examine the frequency of phase variation and the role of these phase variable systems in conditions mimicking human infection.

## Materials and methods

Serum was isolated from human blood from healthy volunteers with informed written consent (in accordance with the guidelines and approval of the Griffith University Human Ethics Committee (HREC 2012/798)).

### Bioinformatic identification of SSRs

Sequences of five closed *M*. *catarrhalis* genomes (BBH18 [53], 25239 [27], 25240 [54], FDAARGOS_213 (Accession NZ_CP020400), and CCRI-195ME [55]) analysed in this study were acquired from GenBank. All possible combinations of repeats of between one and nine repeating units were formulated and the five closed *M*. *catarrhalis* genomes were searched for these sequences, as described previously [56]. Data generated in these analyses were moved into spreadsheets for manual curation, and comparison of repeat lengths in a specific genes between strains was performed using an alignment of the five genomes (aligned using Geneious version 10.1.3 (http://www.geneious.com) [57] with the mauve plug-in [58]). Positive hits were omitted from further analysis if the SSR was found in less than three of the five genomes, varied due to SNP mutations rather than insertion/deletion of a repeat unit, or was located in regions of high frequency recombination (e.g. phage or transposon associated ORFs).

### Bacterial strain and growth conditions

*M*. *catarrhalis* strains used in this study include CCRI-195ME [55], American Type Culture Collection (ATCC; Manassas, VA, USA) strains ATCC 25239 and ATCC 23246, and derivatives thereof (Table 2). *M*. *catarrhalis* strains were grown on brain heart infusion (BHI) agar (Oxoid, Basingstoke, UK) at 37°C with 5% CO_2_, or in BHI broth (Oxoid, Basingstoke, UK) at 37°C with orbital shaking at 200 rpm.

### Fragment length analysis

Fragment length analysis was performed with strains 195ME and 25239 when measuring the length of repeat tracts in the *uspA1*, *uspA2*, *mid/hag*, *gor* (glutathione disulphide reductase), and *hyp* (hypothetical permease MC25239_RS00020), and with strain 23246 for the *modO* gene, as previously described [27, 59]. Briefly, 100–300 bp regions spanning SSRs were amplified with 6-Carboxyfluorescein (6-FAM) or Hexachlorofluorescein (HEX) labelled primers (Integrated DNA Technologies) (S1 Table) using GoTaq polymerase (Promega) as per manufacturer’s instructions. The size and relative quantity of each fluorescently labelled amplicon was measured using a 3130xl Genetic Analyser and GeneScan (Applied Biosystems, Grand Island, NY, USA), and electropherogram traces were visualized using Peakscanner version 1.0 (Applied Biosystems, Grand Island, NY, USA). Selection of *M*. *catarrhalis* subpopulations containing predominantly one repeat tract length for a given gene was carried out by repeated rounds of fragment length analysis and subculturing, as previously described [37].

### Quantitative real time PCR (qRT-PCR)

Overnight plate cultures of *M*. *catarrhalis* were standardised to an optical density of OD_600_ = 0.1 in 20 mL BHI broth and grown for an additional 3.5 hours. 4 mL of RNAprotect Bacteria Reagent (Qiagen) was added to 2mL of bacterial culture, and RNA was extracted using the RNeasy Mini Kit (Qiagen) enzymatic lysis protocol as per manufacturer’s instructions. RNA was subsequently treated with DNaseI (NEB) and the absence of contaminating DNA was confirmed by PCR using GoTaq polymerase (Promega) and *copB* primers (S1 Table). cDNA was prepared using ProtoScript II Reverse Transcriptase (NEB), with random Primer 6 (NEB) and 1 μg RNA. 2.5 ng of cDNA was used as the template in 20 μl qRT-PCR reactions with SsoAdvanced Universal SYBR Green Supermix (Biorad). qRT-PCR reactions were performed in triplicate using a CFX96 Real-Time PCR Detection System (Biorad), and primers for these reactions are listed in S1 Table. The *copB* gene was used as an endogenous reference to normalize the results obtained with the phase variable genes.

### Biofilm formation assays

*M*. *catarrhalis* strains were grown overnight on BHI agar, resuspended in BHI broth, and standardized to OD_600_ = 1. Cells were diluted 1:10 into chemically defined media [60], and 1 mL aliquots were dispensed into wells of a 24-well plate in triplicate (final concentration of approximately 10^7^ CFU/mL of *M*. *catarrhalis*). Plates were incubated at 37°C with orbital shaking at 100 rpm for 24 hours. Media and planktonic cells were then aspirated from wells, and adherent cells were scraped from plates and resuspended in fresh BHI broth. 100 μl of each suspension was used to inoculate 900 μl of freshly prepared chemically defined media in a new 24-well plate, with passaging performed for three consecutive days.

### Serum survival assays

Serum was isolated from human blood from volunteers, using Bio-One Vacuette serum separator tubes (Greiner) and processed as per manufacturer’s instructions. *M*. *catarrhalis* strains were grown overnight on BHI agar, standardized to OD_600_ = 0.05 in 20 mL BHI broth, and grown for a further 3.5 hours to mid-log phase. Cultures were equalised to OD_600_ = 1.0 in fresh BHI broth, three 10-fold serial dilutions were performed, and 10 μl of this suspension was inoculated into 90 μl serum in triplicate in a 96-well plate (final concentration of approximately 10^4^ CFU/mL of *M*. *catarrhalis*). Due to differences in innate serum resistance, strain CCRI-195ME was incubated in 90% serum, and strain 25239 was incubated in 10% and 20% serum. Plates were incubated at 37°C, 5% CO_2_, and after 24 hours of growth, 10 μl was transferred to a new plate of serum, with passaging performed for 3 consecutive days.

## Results

### Bioinformatic identification of phase variable repeat tracts in *M*. *catarrhalis*

The closed genomes of five *M*. *catarrhalis* strains (BBH18, 25239, 25240, FDAARGOS_213 and CCRI-195ME) were analysed to identify SSRs and genes associated with them. Only SSRs containing greater than 7 mononucleotide (e.g., G_(n)_), 3 dinucleotide (e.g., AT_(n)_), or 2 trinucleotide, tetranucleotide, or pentanucleotide (e.g., TAA_(n)_, CAAC_(n)_, CAACG_(n)_), repeat units were included, as these represent the minimum number of repeat units likely to allow phase variation [38]. To compare repeat tracts from the five genomes, the location of repeat tracts relative to reading frames (upstream, within or downstream) was noted, and the repeat-associated genes were considered the same ORF if the BLASTn reported P value was less than 10^−3^. The dataset was further curated by review of repeat sequences to ensure tracts from different genomes were grouped under common designations (e.g. the repeat unit in the site-specific DNA methyltransferase *modM* was listed as AACC and ACCA in different genomes, but generates the same overall repeat tract sequence). At this stage, repeat tracts were removed from the analysis if all five genomes contained the same number of repeats in the same relative position, as this indicated that the repeats are not phase variable. After this process, 212 putative phase variable repeat tracts were identified across the five genomes.

To allow visual inspection of repeat regions, genomes were aligned with Mauve in Geneious. From this, repeat tract duplications were removed (i.e., if the same repeat tract had been associated with the genes upstream and downstream of it) and any missing data from specific strains was added (e.g. repeats may not have been included in the dataset if repeat numbers fell below set thresholds in a genome for that strain). From these data, a shortlist of repeat tracts that were likely to be phase variable was formed based on whether there was sufficient data available to assess if a region was phase variable, and whether the variations in repeat numbers looked to be genuine. In the first case, repeat tracts were eliminated from further consideration if the associated ORF was not present in at least three of the five genomes, or if the repeat tracts were present in regions with markers of high frequency recombination (e.g. phage or transposon related reading frames). In the latter case, repeat sequences were examined, and eliminated from further consideration if repeat tract numbers varied due to single nucleotide polymorphisms in the sequence rather than insertion/deletion of a repeat unit. For single nucleotide repeats, if variability was only seen in one genome or maximum repeat length was at the low end of minimal repeat tracts previously demonstrated to phase vary (e.g. 7 or 8 repeats seen only), tracts were also omitted from further consideration. These analyses left 17 putative phase variable repeat tracts (Table 1). All four genes that have been previously reported to be phase variable (*mid/hag*, *modM*, *uspA1* and *uspA2*) were identified, providing suitable validation for our methodology and thresholds. Three loci (restriction endonuclease subunit M, glutathione disulphide reductase (*gor*), and a hypothetical permease MC25239_RS00020 (*hyp*)) contained upstream repeat tracts which we considered as highly likely to mediate phase variation, as repeat tract lengths varied substantially between the five genomes. However, the restriction endonuclease subunit M was previously found to be a truncated ORF located in a recombinational hotspot for DNA restriction-modification systems and was excluded from further analysis [29]. Although the Type III DNA methyltransferase gene, *modO* (Table 1), was not identified in our bioinformatic analyses as there are no closed RB2/3 lineage genomes, it was included based on our previous work [29].

**Table 1:** Bioinformatic identification of genes with simple sequences repeats in *Moraxella catarrhalis*.

Gene product	Locus tag*	Repeat unit	Repeat location^†^	Number of repeat units				
				195ME	213	BBH18	25239	25240
Mid/Hag	RS03030	G	ORF	6	7	9	10	8
UspA1	RS05840	G	Upstream (29 bp)	11	6	9	8	8
UspA2	RS01775	AGAT	Upstream (132 bp)	14	13	-	18	-
UspA2H*	RS01540	A	ORF	-	-	9	-	-
ModM	RS01915	CAAC	ORF	35	24	29	18	-
ModO*^	McaC031IP	CAACG	Upstream	-	-	-	-	-
Glutathione disulfide reductase	RS01450	GACTGTTT	Upstream (25 bp)	6	17	4	2	3
Hypothetical permease	RS00020	GTTC	Upstream (68 bp)	9	7	18	27	6
Restriction endonuclease subunit M	RS08500	GCGTCAA	Upstream (122 bp)	5	28	2	12	11
RNA methyltransferase	RS02110	TTATCAT	Upstream (14 bp)	1	9	2	1	1
U32 family peptidase	RS02475	TAAAT	Upstream (85 bp)	3	4	3	2	2
Superoxide dismutase	RS03100	T	Upstream (144 bp)	7	6	8	9	7
Hypothetical protein	RS07880	AC	ORF	5	3	4	4	4
Endonuclease III	RS04115	AAACTAT	Upstream (58 bp)	4	3	2	2	3
Molybdopterin biosynthesis protein MoeB	RS03700	T	Upstream (167 bp)	7	7	9	7	6
Magnesium and cobalt transport protein CorA	RS04085	A	Upstream (155 bp)	8	9	7	8	7
Arginase	RS00110	T	Upstream (230 bp)	7	8	9	8	8

Phase variable gene candidates examined in this study are shaded grey.

* All locus tags provided are from *M*. *catarrhalis* strain 25239, except UspA2H (strain BBH18) and ModO (strain C031).

^†^ Position of the repeat sequence is given with respect to the gene start codon consensus in genome comparisons. ORF, open reading from. ORFs and associated SSRs not present in a strain are represented by ‘-‘.

^ *modO* with 12–33 repeat units was identified in eight additional genome strains (C10, C031, N1, N12, R4, Z18, Z7542, and Z7574).

### Differences in numbers of repeat units in phase variable repeat tracts correlate with mRNA transcript level differences for some genes in *M*. *catarrhalis* 25239, CCRI-195ME and 23246

**Fig 1:**
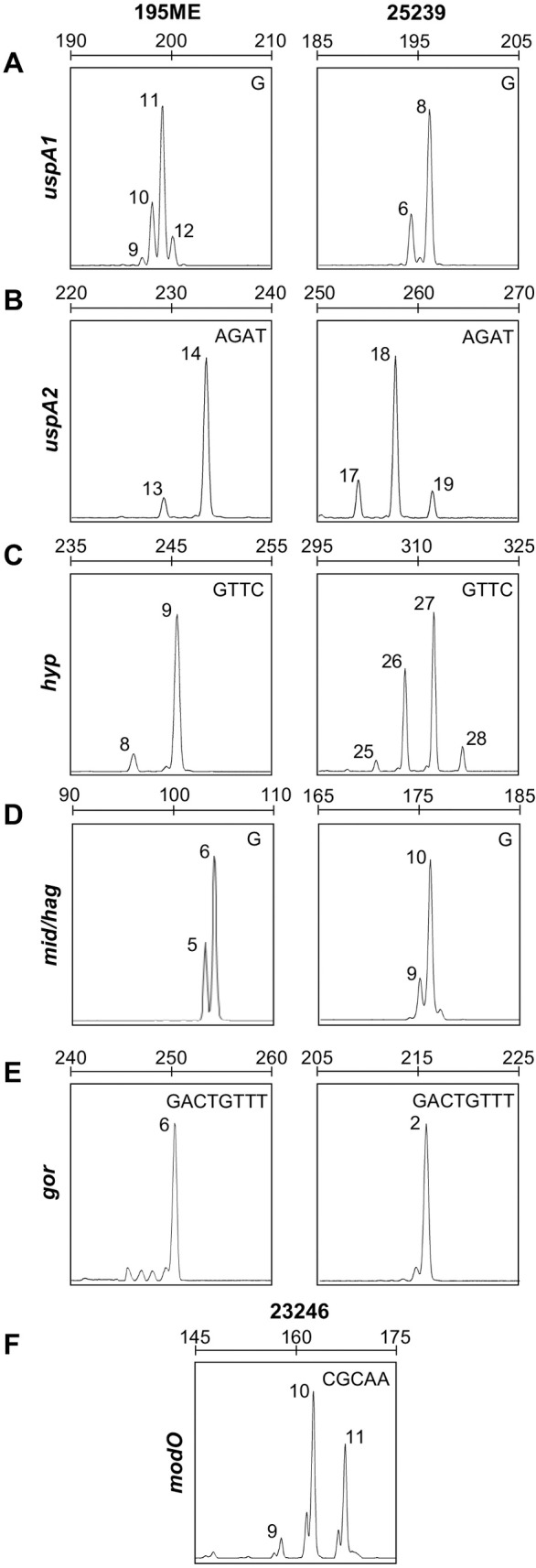
Natural variation in *M*. *catarrhalis* 25239, CCRI-195ME and 23246 DNA repeat tract lengths. Fragment length analysis electropherograms of **A)** *uspA1*, **B)** *uspA2*, **C)** hypothetical permease MC25239_RS00020, **D)** *mid/hag*, **E)** glutathione disulphide reductase gene *gor*, and **F)** *modO* for strains as indicated above. The repeat unit for each gene is shown in the top right corner of each panel. Peaks are labelled individually with the number of repeat units present in each amplicon. Scale bars indicate amplicon length in base pairs.

Six loci were selected to characterise phase variation in detail–the three previously identified phase variable loci (*mid/hag*, *uspA1*, *uspA2*) and three novel loci with a high likelihood of being phase variable (*hyp*, *gor* and *modO*). The length of the DNA repeat tracts in these genes, and the percentage of each repeat length in a population was determined by GeneScan fragment length analysis of SSR amplicons. Initial screening of groups of 12 pooled *M*. *catarrhalis* 25239 and 195ME single colonies revealed the presence of multiple *uspA1*, *uspA2*, *mid/hag*, and *hyp* amplicons of varying size, corresponding to natural variation in the number of repeat units in each gene’s SSR tract (Fig 1A–1D). Screening of *M*. *catarrhalis* 23246 colonies similarly demonstrated that natural variation in repeat tract length occurs in the *modO* gene (Fig 1F). No variation in *gor* glutathione disulphide reductase amplicon size was observed in *M*. *catarrhalis* 25239 or 195ME, suggesting that *gor* is not phase variable under standard laboratory conditions, or the frequency of *gor* phase variation is too low for SSR variants to be detected with our method (Fig 1E). Through successive passaging and fragment length analysis of single colonies derived from these initially mixed populations, *uspA1*, *uspA2*, *hyp* and *modO* SSR variants were isolated that were highly enriched for repeat tracts of a single length (Table 2). Although the poly-G tract of *mid/hag* showed natural variation (Fig 1D), populations with enriched repeat tract lengths could not be successfully isolated after several rounds of enrichment.

**Table 2:** Summary of fragment length analysis amplicon size, DNA repeat number and relative expression of phase variable gene panel populations used in this study.

Strain	Gene	Amplicon size (bp)^a^	# of repeats	Abundance^b^	Relative mRNA ratio (p-value)^c^	Relative mRNA level^d^
**195ME**	*uspA1*	196	8	83%	1.44 (0.36)	Low
		197	9	83%	2.94 (0.11)	Low
		198	10	70%	1.00	Low
		199	11	66%	19.83 (0.0016)	High
		200	12	55%	21.31 (0.0039)	High
	*uspA2*	229	12	64%	1.00	Low
		233	13	95%	1.24 (0.025)	Mid
		237	14	92%	1.44 (0.0085)	High
		241	15	94%	1.43 (0.0060)	High
		245	16	90%	1.22 (0.067)	Low
	*hyp*	241	8	98%	1.45 (0.0068)	High
		245	9	98%	1.00	Low
**25239**	*uspA2*	237	14	95%	1.89 (0.00048)	Mid
		241	15	93%	1.86 (0.00052)	Mid
		245	16	87%	2.59 (0.0004)	High
		249	17	94%	3.37 (0.00043)	High
		253	18	92%	1.09 (0.15)	Low
		257	19	91%	1.00	Low
		261	20	91%	1.28 (0.0083)	Mid
	*hyp*	308	25	91%	1.00	Low
		312	26	90%	1.20 (0.025)	High
		316	27	89%	1.02 (0.53)	Low
**23246**	*modO*	157	9	92%	1.00	Low
		162	10	81%	20.66 (0.0000088)	High
		167	11	98%	1.75 (0.000014)	Mid

^a^ Fragment length analysis amplicons were generated using primers directed against conserved regions outside the SSR region (as described in S1 Table), and calibrated for size using synthetic Geneblocks (IDT) of known size where necessary.

^b^ % Abundance is the proportion of the stock population expressing the specified fragment length analysis amplicon size, as assessed by fragment length analysis.

^c^ The relative mRNA ratio indicates the mRNA level of each phase variant relative to the variant with the lowest detected mRNA level (set as 1.00). P-values were calculated using a two-tailed Student’s *t-*test comparing mRNA levels relative to the variant with the lowest mRNA.

^d^ The relative mRNA level is arbitrarily classified as low, mid or high based on the magnitude and statistical significance of difference between variants, and is included for ease of comparison between variants.

**Fig 2:**
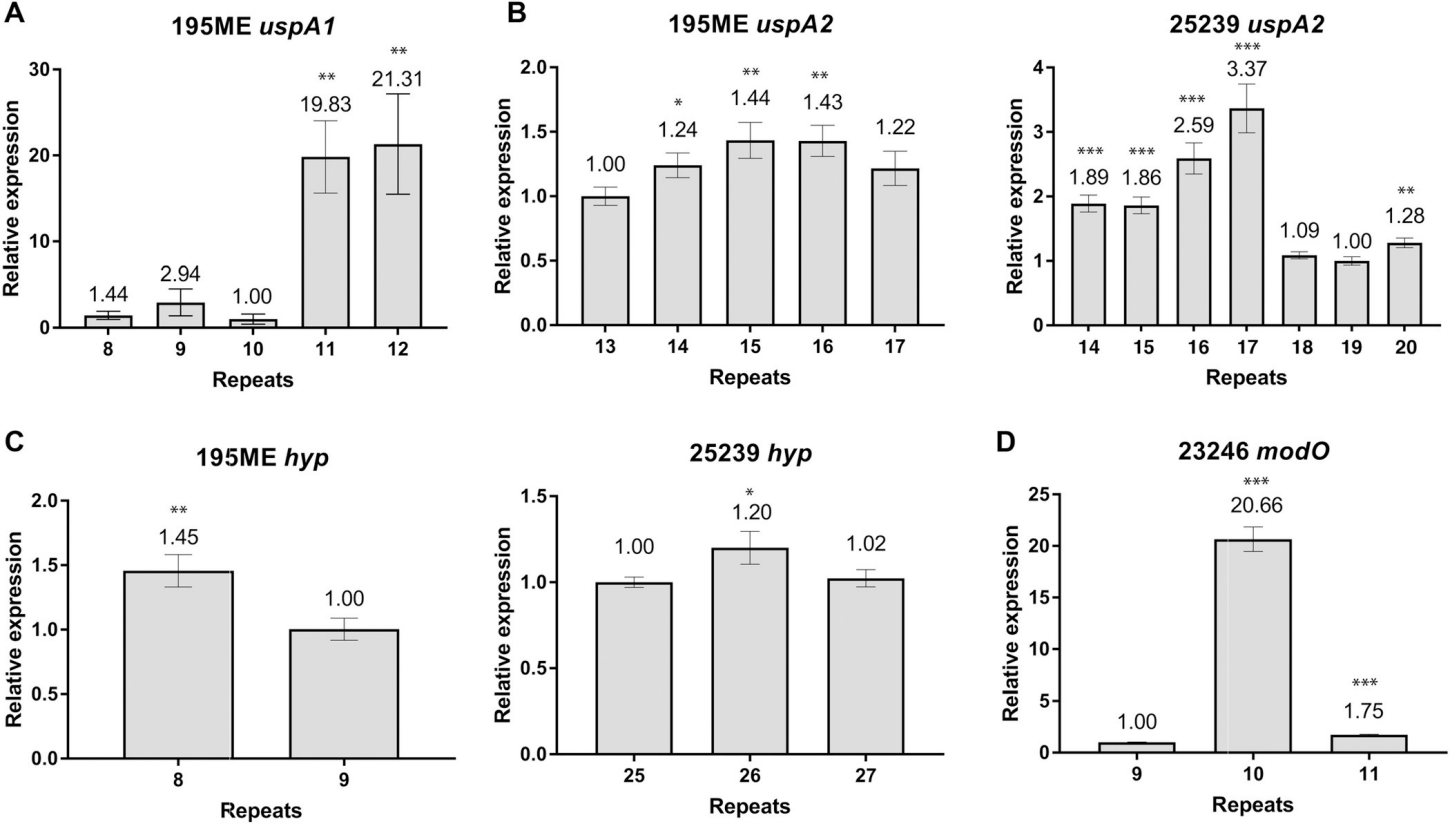
Gene transcript levels relative to DNA repeat tract sizes. Relative differences in mRNA levels measured by qRT-PCR for **A)** *uspA1*, **B)** *uspA2*, **C)** hypothetical permease MC25239_RS00020, and **D)** *modO*. Fold-change is calculated relative to the variant with the lowest expression (set as 1.00), and values are shown above the bars. P-values were calculated using a two-tailed Student’s t-test comparing mRNA levels relative to the variant with the lowest mRNA (set at 1.00). *, P < 0.05 **, P ≤ 0.01, ***, P ≤ 0.001.

To determine whether variations in SSR tract length correspond to differences in detectable mRNA transcript levels, we selected variants with defined SSR lengths (as determined by fragment length analysis) and compared the mRNA levels of *uspA1*, *uspA2*, *hyp* and *modO* by qRT-PCR. For *uspA1* in strain 195ME, changes in SSR resulted in substantial changes in mRNA levels, with G_(n)_ tract lengths containing 8, 9 or 10 G residues corresponding to low mRNA, and 11 or 12 G residues corresponding to high mRNA (21.31 fold difference between 10 G residues and 12 G residues; Fig 2A). *uspA1* expression was not investigated in *M*. *catarrhalis* 25239, as this strain contains nonsense mutations in the *uspA1* gene. *uspA2* shows a maximum difference in mRNA levels of 3.37-fold between 17 and 19 5′-AGAT_(n)_-3′ repeat units in *M*. *catarrhalis* 25239. However, only a modest difference in expression is observed between 12–16 5′-AGAT_(n)_-3′ repeat units in strain 195ME (Fig 2B), and isolation of variants with longer repeat tracts may be required for maximal differences in expression to be observed in this strain. The number of 5′-GTTC_(n)_-3′ repeats in the *hyp* repeat tract minimally affected MC25239_RS00020 mRNA levels, with a 1.43 fold and 1.2 fold difference in expression observed in strains 195ME and 25239, respectively (Fig 2B). However, only a limited number of *hyp* SSR variants could be isolated in this study and further analysis using a broader range of repeat variants is required to adequately assess *hyp* phase variation. In contrast, mRNA levels of *modO* in *M*. *catarrhalis* 23246 was clearly correlated with repeat number; low levels of *modO* mRNA was observed when 9 or 11 5′-CAACG_(n)_-3′ repeats are present, while 11.81–20.66 greater mRNA was observed when 10 5′-CAACG_(n)_-3′ repeats were present (Fig 2D).

### Expression of the adhesins encoded by *uspA1* and *mid/hag* are selected against during biofilm formation

**Fig 3:**
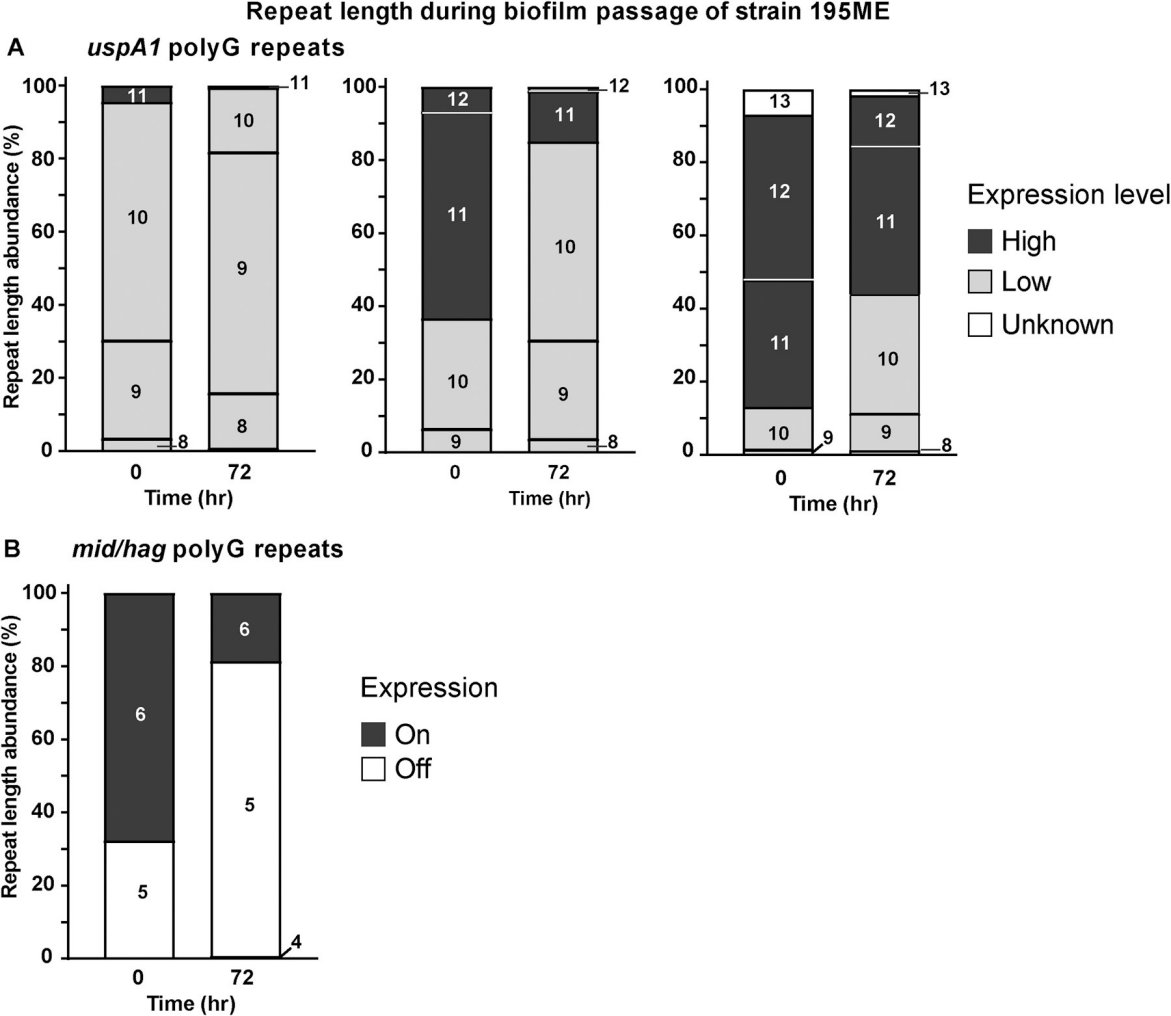
Differences in *uspA1* and *mid/hag* repeat tract lengths during biofilm passaging. Fragment length analysis of *M*. *catarrhalis* 195ME populations passaged in biofilm formation assays for 3 consecutive days. Each graph shows a different starting population, enriched for **(A)** 10, 11 or 12 repeats in *uspA1*, or **(B)** 6 repeats in *mid/hag*. Stacked bars indicate the proportion of each repeat length found in pre-passaging (0 hrs) and post-passaging (72 hrs) populations. For *uspA1*, bar colour indicates the relative mRNA level correlated with each repeat length: black, high mRNA level; grey, low mRNA level; white, unknown mRNA level (*uspA1* 13 repeat variant only). For *mid/hag* bar colour indicates expression phase: black, on; white, off. Assays were carried out in biological triplicate, and proportions averaged. See S1 Fig for individual assays.

Phase variable expression of *mid/hag*, *modM*, *uspA1*, *uspA2*, *gor* and *hyp* were next analysed in conditions mimicking stages of *M*. *catarrhalis* human infection. Biofilms are multicellular communities of bacteria that are frequently seen in colonisation of hosts and that are often responsible for increased antibiotic resistance and persistent infections [61, 62]. Bacterial cells have a series of surface adhesins that aid biofilm formation [63] and *M*. *catarrhalis* has been identified in biofilms in tube otorrhea [64] and middle ear effusions [65]. Therefore, we passaged strains under biofilm forming conditions to identify whether this selected for strains with enriched phase variants. For this work, we started with three separate *M*. *catarrhalis* 195ME populations with different proportions of SSRs in *uspA1* (i.e., with starting populations enriched for low expression (G_10_ in the SSR) or high expression (G_11_ or G_12_)). Regardless of the initial repeat tract length, after three rounds of selection of biofilm cells all resultant populations are significantly skewed to repeat tract lengths correlating to low levels of *uspA1* expression (G_10_ residues) (Figs 3A and S1). Expression of *mid/hag* is also selected against in biofilm selection, with a distinct shift in SSR length that results in an on to off switch of mRNA expression (Figs 3B and S1). No consistent shift of greater than 10% of the population was observed for *modM*, *uspA2*, *gor*, or *hyp* repeat tract lengths during biofilm selection these samples (S1 Fig).

### High *uspA2* expressing variants are selected for during growth in human serum

**Fig 4:**
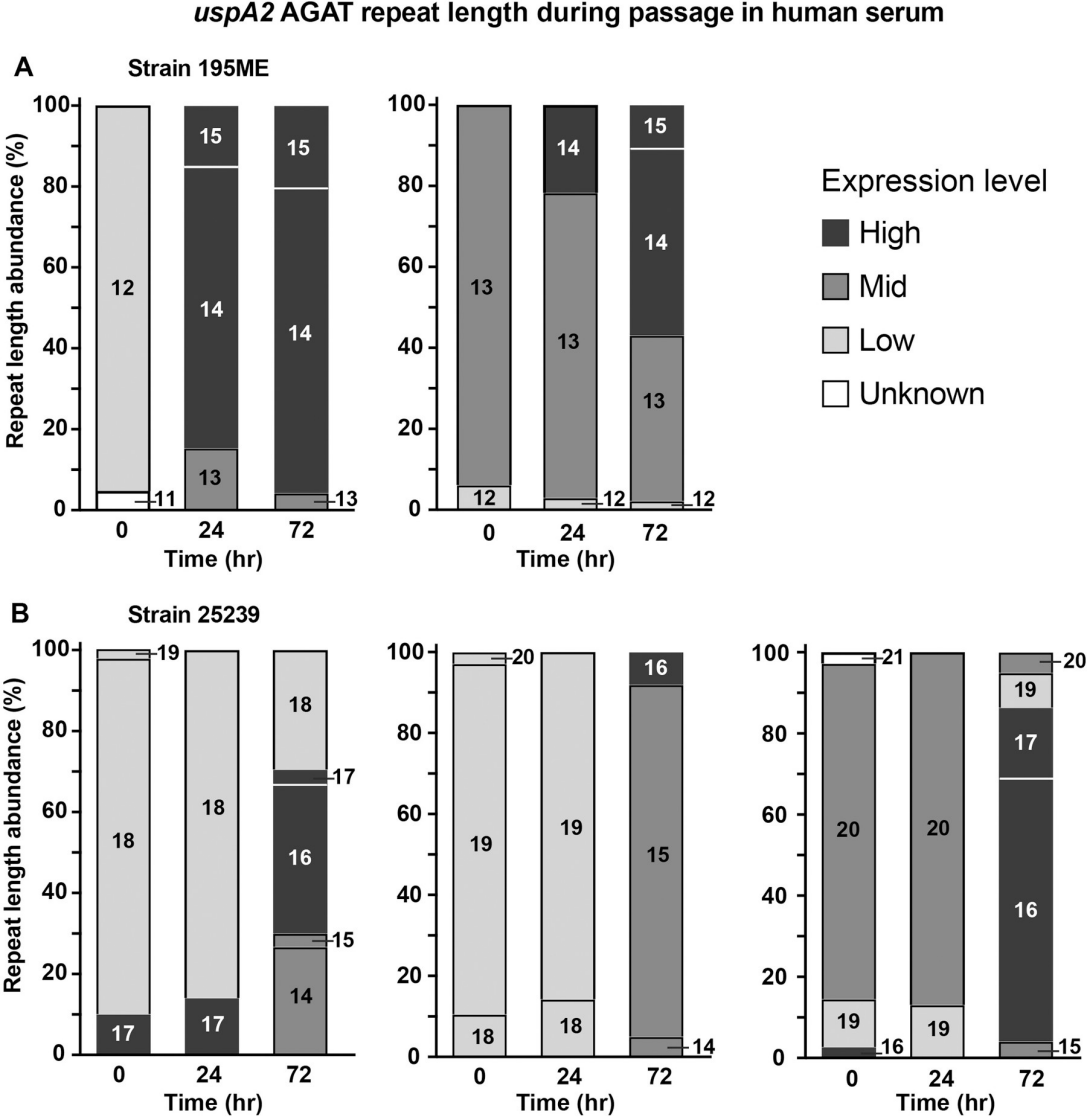
Differences in *uspA2* repeat tract lengths during serum passaging. Fragment length analysis of *M*. *catarrhalis* 195ME and 25239 populations passaged in serum for 3 consecutive days. Each graph shows a different starting population, enriched for **A)** 12 or 13 repeats in *uspA2* in strain 195ME, or **B)** 18, 19, or 20 repeats in strain 25239. Stacked bars indicate the proportion of each repeat length found in pre-passaging (0 hrs) and post-passaging (24 or 72 hrs) populations. Bar colour indicates the relative expression level correlated with each repeat length: black, high expression; dark grey, mid expression; light grey, low expression; white, unknown expression level. Assays were carried out in biological triplicate, and proportions averaged. See S2 Fig for individual assays.

Complement‐mediated killing is an important aspect of the human innate immune response, with increased levels of complement factors present during OM and exacerbations of COPD, Serum resistance is considered a key virulence factor of *M*.* catarrhalis* [66], and UspA2 is involved in serum resistance in many strains [67]. Therefore, serum killing assays were performed with *M*. *catarrhalis* strains 25239 and 195ME inoculated into human serum and grown for 24 hours, and serially passaged for three days to determine if serum effected selection of *mid/hag*, *modM*, *uspA1*, *uspA2*, *gor*, *hyp* expression. Selection for high level expression of *uspA2* occurred in both strain 195E and 25239 after three passages in human serum (Figs 4 and S2). Specifically, starting with populations enriched for low or mid *uspA2* expression (i.e., *uspA2* SSR variants with 12–13 A_(n)_ repeats for strain 195ME, and 18–20 repeats for strain 25239), serum selection resulted in populations enriched for SSRs consistent with high level expression (195ME enriched for tract lengths of 14–15 A_(n)_ and strain 25239 was enriched for SSR tract lengths of 15–17 A_(n)_). No consistent, significant or substantial shift was seen in SSR tract length for other putative phase variable determinants in this assay (S2 Fig).

## Discussion

Phase variation is the high frequency reversible switching of gene expression, which can provide a selective advantage for bacteria as expression of determinants can be temporarily altered without changes to the coding sequence of the gene [13]. As a result, phase variation allows cells to evade specific antibodies and generate population heterogeneity. In our bioinformatic analysis of five *M*. *catarrhalis* genomes, we identified 17 unique SSRs with the potential to mediate phase variable expression of the associated genes. Of those, we observed natural variation in SSR in the genomes of *M*. *catarrhalis* for five genes. We found that altered SSR tract lengths modulates levels of mRNA transcript detectable by qRT-PCR, and that phase variation of three major outer membrane proteins occurs due to selection pressure exerted under biologically relevant conditions.

Our work details that phase-variable expression of the *uspA1*, *uspA2*, and *mid/hag* genes is associated with changes in SSR repeat tract lengths, confirming earlier findings for *uspA1* [20], *uspA2* [21], and *mid/hag* [23]. In addition to being phase-variably expressed, *uspA1* and *uspA2* show significant sequence variation (antigenic variation) between strains due to rearrangement, deletion, and duplication of modular domains [20, 23, 68]. This variability is concentrated in surface-exposed regions, while their membrane-embedded regions are more conserved [68]. Antigenic variation is a feature of many outer-membrane proteins in various host adapted pathogens and aids in immune-evasion [49]. In addition, two further variants of UspA2 have been described: the hybrid UspA1-UspA2 protein known as UspA2H [16] and UspA2V [69]. The consequence of combining both phase and antigenic variability in a single gene/gene product is a high degree of strain to strain variability. Furthermore, when sequence variability is located in genomic regions near SSRs, this variability may also complicate the analysis of expression. For example, while it is reported that in strains O12E and O35E *uspA1* repeat tracts of G_(9)_ and G_(10)_ cause low and high level expression, respectively [20], we found that in strain 195ME high *uspA1* mRNA levels are associated with G_(11)_ and G_(12)_ tract lengths. Sequence analysis of the *uspA1* gene in these three strains shows this difference is likely due to the presence of an additional adenine residue upstream of the repeat region in strain 195ME. Similarly, while expression of the *uspA2* locus has been reported to correlate with increasing numbers of 5′-AGAT_(n)_-3′ repeats in strain O12E, with maximal expression seen with 18 5′-AGAT_(n)_-3′ repeats [21], we found that 14–15 and 16–17 5′-AGAT_(n)_-3′ repeat units correspond to the highest levels of mRNA transcripts in strains 195ME and 25239, respectively. This emphasises the effect that variability between genomes may have on expression levels, and should serve as a caveat for future studies. Given the phase variability of the UspA1, UspA2 and Mid/Hag proteins and necessity of determining expression status on a case-by-case basis, these proteins would not necessarily be considered to be good vaccine candidates. However, these proteins are major outer membrane components of *M*. *catarrhalis* and comprise most of the surface projections from the cell [22]. They also mediate key virulence pathways, including biofilm formation [15] and adherence to multiple epithelial cell types [16, 17] and extracellular matrix components [18, 19] for UspA1; serum resistance and binding to vibronectin [21] and agglutination of *M*. *catarrhalis* cells, red blood cells and immunoglobulin D [22, 23] for Mid/Hag. In this case, it may still be possible to use these determinants as vaccine candidates if they remain expressed over the course of an infection for targeting by the immune system. However, our work showed that expression of *uspA1* and *mid/hag* is not stable over long periods under conditions mimicking human infection, with expression decreasing over time in biofilms on an abiotic surface. A recent report also found that *mid/hag* expression is turned off in clinical samples during infection [24]. This suggests that Mid/Hag in particular is not a good vaccine candidate, as its expression is not required for persistent colonisation. In contrast, expression of *uspA2* increases over time in serum and may provide a more consistent vaccine target during infection. It is interesting to note that there is no variation of *mid/hag* and *uspA1* during exposure to human serum, as compared to the biofilm passaging, whereas *uspA2* shows variation under exposure to serum but not during biofilm passaging. These data suggest that whilst individual cells in a population may phase vary at random, the overall population maintains a relatively stable proportion of variants unless a selective force is present and a difference in fitness between variants exists.

This study demonstrated that the *modO* gene is phase variable due to 5ʹ-CAACG_(n)_-3ʹ repeats upstream of its ORF, and that there is a 20-fold difference in detectable transcript of *modO* depending on repeat tract size. The confirmation of the phase variable nature of *modO* brings the number of phase variable DNA methyltransferases in *M*. *catarrhalis* to three (*modM*, *modN* and *modO*), and transcriptomic or proteomic analysis will be required to confirm whether ModO regulates a phasevarion in *M*. *catarrhalis*. Investigation of how these epigenetic regulators interact with other virulence determinants of *M*. *catarrhalis* may provide valuable insight into the pathogenicity of this species.

The length of the SSR tract associated with the MC25239_RS00020 hypothetical permease clearly shows variation between strains and within individual strains that have been serially passaged, however substantial differences in transcription levels were not observed under the conditions tested. Although a large range of *hyp* SSR repeats were observed between strains (6–27 5'-GTTC_(n)-_3' repeats), only a few *hyp* SSR variants were isolated. Greater differences in expression may be observed upon isolation of the full range of SSR lengths, and this warrants further investigation. In the case of the glutathione sulphide reductase gene, *gor*, inter-genome variation in SSR tract length does not appear to be correlated with phase variation as no natural variation in *gor* repeat length was observed within an individual strain. However, future studies of *gor* using a strain with a higher number of repeats within the 5'-GACTGTTT_(n)-_3' SSR tract, such as the 17 repeat tract in strain FDAARGOS_213, may allow phase variation to be observed at a measurable frequency. Further investigations into these two loci are needed to fully elucidate if these genes are phase-variably expressed, and determine their role in *M*. *catarrhalis* pathobiology.

Relative to other respiratory tract pathogens, *M*. *catarrhalis* does not appear to have as large a number of phase variable genes that vary by SSRs. However, the phase variable genes identified in this study may not represent the full repertoire of phase variable genes in *M*. *catarrhalis* due to several limitations of our analysis. Firstly, screening was restricted to only the five closed genomes available at the time of analysis (necessitated as short-read assemblies often split SSRs between contigs, preventing complete analysis of SSR length). In addition, all of the five closed genomes analysed are from the *M*. *catarrhalis* RB1 lineage, and consequently, phase variable genes specific to the RB2/3 lineage may remain to be identified–as we found with *modO* which was identified in our previous work [29]. Phase variable genes may have been omitted from analysis due to our strict criteria. For example, repeat tracts were eliminated from further consideration if they and the associated reading frames were not present in a majority of the genomes, or if only minimal repeat variation was seen (particularly with polynucleotide SSR). These exclusions do not reflect on the potential variability of these tracts, but on the diversity of genomes used. In addition, phase variation can also occur via other mechanisms, for example genome inversion as seen with *fimS* in *E*. *coli* [70], or site-specific recombination as observed for Type I restriction-modification systems [71]. However, identification of genes that switch expression by these mechanisms is complicated, due to their mediating motifs often being short and highly variable in sequence, placement, and orientation, and was not within the scope of this study.

Overall, we define the repertoire of simple sequence repeats and putatively phase variable genes in *M*. *catarrhalis*. In addition, we show that phase variation of several genes occurs during growth and in conditions that mimic human infection which contributes to our understanding of *M*. *catarrhalis* pathogenesis and will aid in future selection of candidate vaccine antigens.

## Supporting information

S1 Fig Analysis of DNA repeat tract lengths of putative phase variable genes during biofilm passaging. Fragment length analysis of *M*. *catarrhalis* 195ME populations passaged in biofilm formation assays for 3 consecutive days. Each graph includes three different starting populations, enriched for 10, 11 or 12 repeats in *uspA1* (Sample 1, 2, or 3, respectively). Assays were carried out in triplicate, and each circle indicates a separate repeat (closed circle is at 0 h; open circle is at 72 h). The bar represents the mean, and error bars represent ±1 standard deviation. A two-tailed Student’s *t-*test was used to compare time 0 h vs 72 h (*, P < 0.05 **, P ≤ 0.01, ***, P ≤ 0.001).
(PDF)

S2 Fig Analysis of DNA repeat tract lengths of putative phase variable genes during serum passaging. Fragment length analysis of *M*. *catarrhalis* 195ME and 25239 populations passaged in serum for 3 consecutive days. Each graph includes five different starting populations, enriched for 12 or 13 repeats in *uspA2* in strain 195ME (Sample 1 or 2, respectively) or 18, 19, or 20 repeats in *uspA2* in strain 25239 (Sample 1 or 2, or 3, respectively). Assays were carried out in triplicate, and each circle indicates a separate repeat (closed circle is at 0 h; open circle is at 72 h). The bar represents the mean, and error bars represent ±1 standard deviation. A two-tailed Student’s *t-*test was used to compare time 0 h vs 72 h (*, P < 0.05 **, P ≤ 0.01, ***, P ≤ 0.001).
(PDF)

S1 Table Primers used in this study. (PDF)
