# Supplementary material for: Moraxella catarrhalis phase-variable loci show differences in expression during conditions relevant to disease
Source: PLoS One. 2020 Jun 18;15(6):e0234306. doi: 10.1371/journal.pone.0234306 (PMC7302503; doi:10.1371/journal.pone.0234306)
Supplement: S1 Fig — Fragment length analysis of M. catarrhalis 195ME populations passaged in biofilm formation assays for 3 consecutive days. Each graph includes three different starting populations, enriched for 10, 11 or 12 repeats in uspA1 (Sample 1, 2, or 3, respectively). Assays were carried out in triplicate, and each circle indicates a separate repeat (closed circle is at 0 h; open circle is at 72 h). The bar represents the mean, and error bars represent ±1 standard deviation. A two-tailed Student’s t-test was used to compare time 0 h vs 72 h (*, P < 0.05 **, P ≤ 0.01, ***, P ≤ 0.001). (PDF) [file pone.0234306.s001.pdf]

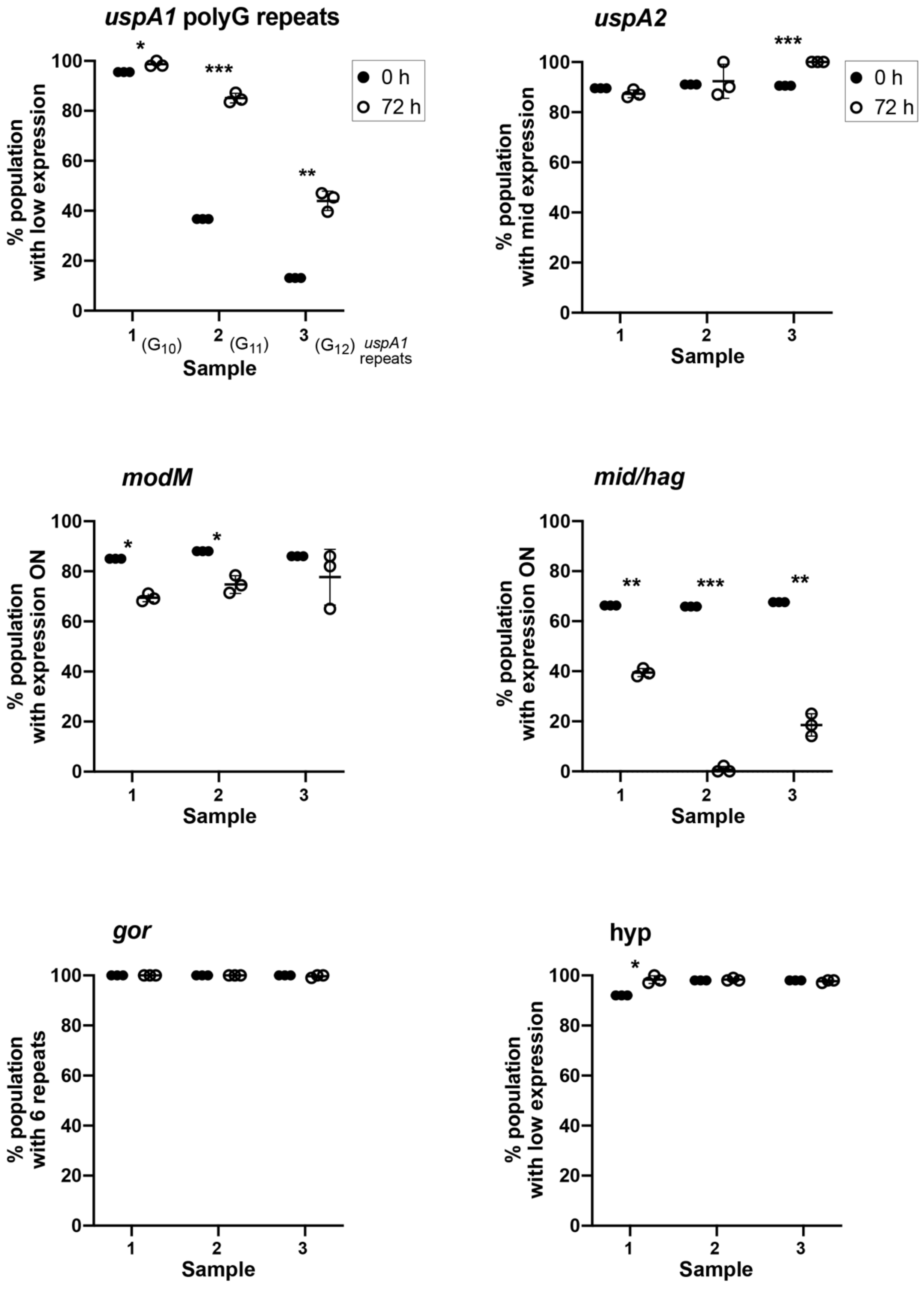

S1 Fig. Analysis of DNA repeat tract lengths of putative phase variable genes during biofilm passaging. Fragment length analysis of *M. catarrhalis* 195ME populations passaged in biofilm formation assays for 3 consecutive days. Each graph includes three different starting populations, enriched for 10, 11 or 12 repeats in *uspA1* (Sample 1, 2, or 3, respectively). Assays were carried out in triplicate, and each circle indicates a separate repeat (closed circle is at 0 h; open circle is at 72 h). The bar represents the mean, and error bars represent ±1 standard deviation. A two-tailed Student's *t*-test was used to compare time 0 h vs 72 h (*, $P < 0.05$ **, $P \leq 0.01$, ***, $P \leq 0.001$).
