# Supplementary material for: Moraxella catarrhalis phase-variable loci show differences in expression during conditions relevant to disease
Source: PLoS One. 2020 Jun 18;15(6):e0234306. doi: 10.1371/journal.pone.0234306 (PMC7302503; doi:10.1371/journal.pone.0234306)
Supplement: S2 Fig — Fragment length analysis of M. catarrhalis 195ME and 25239 populations passaged in serum for 3 consecutive days. Each graph includes five different starting populations, enriched for 12 or 13 repeats in uspA2 in strain 195ME (Sample 1 or 2, respectively) or 18, 19, or 20 repeats in uspA2 in strain 25239 (Sample 1 or 2, or 3, respectively). Assays were carried out in triplicate, and each circle indicates a separate repeat (closed circle is at 0 h; open circle is at 72 h). The bar represents the mean, and error bars represent ±1 standard deviation. A two-tailed Student’s t-test was used to compare time 0 h vs 72 h (*, P < 0.05 **, P ≤ 0.01, ***, P ≤ 0.001). (PDF) [file pone.0234306.s002.pdf]

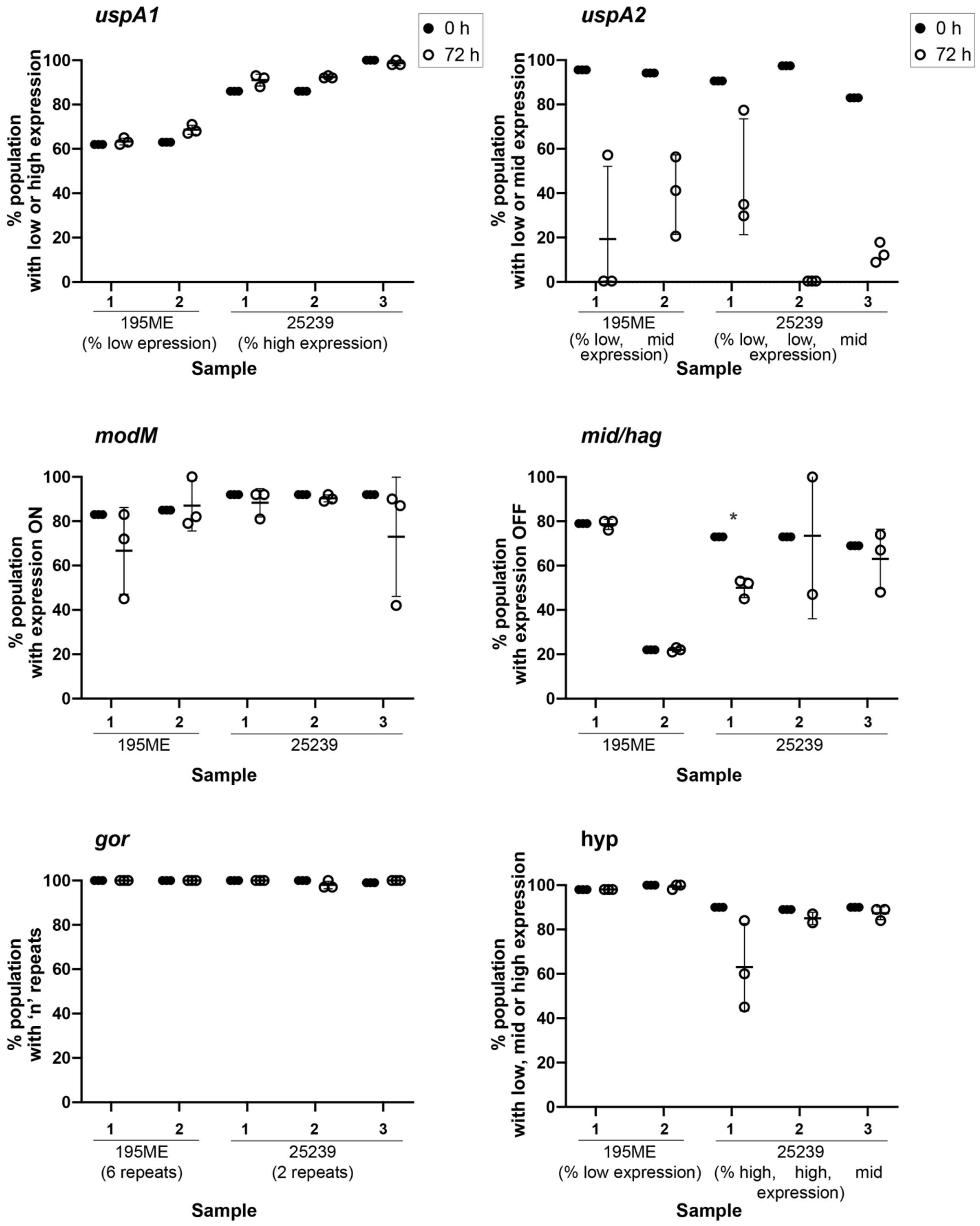

S2 Fig. Analysis of DNA repeat tract lengths of putative phase variable genes during serum passaging. Fragment length analysis of *M. catarrhalis* 195ME and 25239 populations passaged in serum for 3 consecutive days. Each graph includes five different starting populations, enriched for 12 or 13 repeats in *uspA2* in strain 195ME (Sample 1 or 2, respectively) or 18, 19, or 20 repeats in *uspA2* in strain 25239 (Sample 1 or 2, or 3, respectively). Assays were carried out in triplicate, and each circle indicates a separate repeat (closed circle is at 0 h; open circle is at 72 h). The bar represents the mean, and error bars represent ±1 standard deviation. A two-tailed Student's *t*-test was used to compare time 0 h vs 72 h (*, $P < 0.05$ **, $P \leq 0.01$, ***, $P \leq 0.001$).
