# Supplementary material for: Moraxella catarrhalis phase-variable loci show differences in expression during conditions relevant to disease
Source: PLoS One. 2020 Jun 18;15(6):e0234306. doi: 10.1371/journal.pone.0234306 (PMC7302503; doi:10.1371/journal.pone.0234306)
Supplement: S1 Table — (PDF) [file pone.0234306.s003.pdf]

S1 Table: Primers used in this study

Name	Sequence	Description*
**Fragment length analysis primers**		
[HEX]-gds_F	5'- CCATCCGAGTCAGAGTATTG-3'	HEX-labelled *gor* forward primer
gds_R	5'- CCAATGGCAAGATAGTCATAATG-3'	*gor* FLA reverse primer
[6FAM]-hyp_F	5'-GGTTGGGTATTATTGCTTGATAG-3'	6FAM-labelled *hyp* (MC25239_RS00020) forward primer
hyp_R	5'- AGACCCATCAAAAGTCCTGC-3'	*hyp* (MC25239_RS00020) reverse primer
[6FAM]-Mid/hag F	5'-GTCATCTTTAACAAAGCCACAGG-3'	6FAM-labelled *mid/hag* forward primer
mid/hag_R1	5'-AAAGCTCAGAGTGCGTACAC-3'	CCRI-195ME specific *mid/hag* reverse primer
mid/hag_R2	5'-GTGCTATTAAACATGGCGGTG-3'	25239 specific *mid/hag* reverse primer
[6FAM]-modM_F	5'-TTACTTGACACTCTGAATGGA-3'	6FAM-labelled *modM* forward primer
modM_R	5'-GTATTATGGGCAGTTTTTAGG-3'	*modM* reverse primer
[HEX]-modO_F	TGGCTTGACATCCAAATTTAAGG	HEX- labelled *modO* forward primer
modO_R	GGCAAATAAATGCTCGGTCG	*modO* reverse primer
[6FAM]-UspA1_F	5'-CATTAGCGATGGCATCAAGTTG-3'	6FAM-labelled *uspA1* forward primer
uspA1_R	5'- AGAACATGCCACCAAGTGAC-3'	*upsA1* reverse primer
[HEX]-UspA2_F	5'-TAAAGCCTTGCTATACTGTAACC-3'	HEX-labelled *uspA2* forward primer
uspA2_R	5'-TAAGTCATCACATCAGTCATCAC-3'	*uspA2* reverse primer
**qRT-PCR primers**		
Hypothetical RT F	5'-AAACCAAAGCTGACCTTAATGGC-3'	*hyp* (MC25239_RS00020)
Hypothetical RT R	5'-AATGGCTTGTACCTCGTCGG-3'	
modO_RT_F	5'-TTGACAGTGTGGCGATTAAAGC-3'	*modO*
modO_RT_R	5'-GCGCTTTCAACCATCTCTCC-3'	
uspA1_RT_F	5'-GTAAGTGCTGCCAATACTGATCG-3'	*uspA1*
uspA1_RT_R	5'-TGCTTCACCTTGCTCAATCAAAG-3'	
uspA2_RT_F	5'-AAGCTGCCCTAAGTGGTCTATTC-3'	*uspA2*
uspA2_RT_R	5'-TAGCCAGCACCGATAGCAAC-3'	
copB_RT_F	5'-GTGAGTGCCGCTTTACAACC-3'	Control primers for *copB* (Greiner et al. 2003)
copB_RT_R	5'-TGTATCGCCTGCCAAGACAA-3'	

*HEX, Hexachlorofluorescein; 6FAM, 6-carboxyfluorescein.
